# Bimodular high temperature planar oxygen gas sensor

**DOI:** 10.3389/fchem.2014.00057

**Published:** 2014-08-19

**Authors:** Xiangcheng Sun, Yixin Liu, Haiyong Gao, Pu-Xian Gao, Yu Lei

**Affiliations:** ^1^Department of Chemical and Biomolecular Engineering, University of ConnecticutStorrs, CT, USA; ^2^Department of Materials Science and Engineering, University of ConnecticutStorrs, CT, USA; ^3^Institute of Materials Science, University of ConnecticutStorrs, CT, USA

**Keywords:** NiO nanoparticles, high temperature, oxygen sensing, bimodular, potentiometric, resistance

## Abstract

A bimodular planar O_2_ sensor was fabricated using NiO nanoparticles (NPs) thin film coated yttria-stabilized zirconia (YSZ) substrate. The thin film was prepared by radio frequency (r.f.) magnetron sputtering of NiO on YSZ substrate, followed by high temperature sintering. The surface morphology of NiO NPs film was characterized by atomic force microscope (AFM) and scanning electron microscope (SEM). X-ray diffraction (XRD) patterns of NiO NPs thin film before and after high temperature O_2_ sensing demonstrated that the sensing material possesses a good chemical and structure stability. The oxygen detection experiments were performed at 500, 600, and 800°C using the as-prepared bimodular O_2_ sensor under both potentiometric and resistance modules. For the potentiometric module, a linear relationship between electromotive force (EMF) output of the sensor and the logarithm of O_2_ concentration was observed at each operating temperature, following the Nernst law. For the resistance module, the logarithm of electrical conductivity was proportional to the logarithm of oxygen concentration at each operating temperature, in good agreement with literature report. In addition, this bimodular sensor shows sensitive, reproducible and reversible response to oxygen under both sensing modules. Integration of two sensing modules into one sensor could greatly enrich the information output and would open a new venue in the development of high temperature gas sensors.

## Introduction

Every year, a huge amount of liquid and solid fuels are consumed through the combustion process (Ramamoorthy et al., 2003). Therefore, *in-situ*, real-time monitoring the composition of combustion gases is important for improving combustion efficiency and reducing the emission of pollutants such as CO, NO_x_, etc. (Miura et al., 2000; Fergus, 2007a,b, 2008; Zhuiykov and Miura, 2007). According to a U.S. Department of Energy report, harsh environment sensors, if successfully employed, are predicted to save 0.25 quadrillion BTU/year of energy across all energy-consuming industries (Miura et al., 2000; Akbar et al., 2006). Therefore, there is an urgent need to design fast, sensitive, selective, rugged, and cost-effective high-temperature gas sensors for power/fuel systems. In the past decades, various gas sensors have been developed for monitoring combustion process (Szabo et al., 2003; Zhang et al., 2007; Liu and Lei, 2013). Among them, solid-electrolyte potentiometric, amperometric, and semiconductor oxide sensors have been extensively studied for high temperature environment.

In particular, high temperature potentiometric sensors have played a pivotal role in pollution control through automobile engine management (Azad et al., 1992; Fergus, 2007a). For the equilibrium potentiometric oxygen sensor, the most direct type uses a solid electrolyte that conducts an ion of the species to be detected, such as an oxygen–ion conductor. Equilibrium potential measurements on solid electrolyte-electrode cells enable oxygen measurement via the Nernst law and the sensor voltage signal typically varies with oxygen partial pressure logarithmically. Disadvantages of such potentiometric sensors include unavailable sensing materials for certain gas species and the requirement of a gas-tight seal between reference and working electrode. Recently, mixed potential gas sensor has attracted much attention and been proposed as an alternative to classical potentiometric sensors due to its stability, sensitivity, and selectivity at high temperatures (Fergus, 2007a; Morata et al., 2008; Plashnitsa et al., 2008). When the sensor is exposed to oxygen, sensor response (electromotive force, EMF) is controlled by the difference in kinetics of redox reactions of oxygen at electrode/electrolyte/gas interface (triple phase boundary, TPB) (Li and Kale, 2007; Sekhar et al., 2010). For the mixed potential type sensors, chemical reactions tend toward equilibrium with increasing temperature, so a sensor response that depends on kinetically limited response leads to decreased signals with the increase of temperature. Consequently, mixed potential type sensors are not favorable for application in very high temperature environment.

High temperature sensors can also be operated in the amperometric mode, in which the electrode reaction is caused by an applied potential and the limiting current, as controlled by diffusion through a small orifice or porous media, is measured (Fergus, 2007a). The mode based on stabilized zirconia exhibits several advantages to detect gaseous compounds like O_2_ and NO_x_ under harsh environments (Reinhardt et al., 2000). Linear relations between the measured current and gas concentrations can be achieved. Although, many of fuel economy vehicles use amperometric sensors, physical modification of the diffusion barrier of the sensor is possible to cause problems (Ramamoorthy et al., 2003). In addition, sensor selectivity is a challenge for amperometric sensors, since any species containing the mobile ion can contribute to the current and thus interfere with the sensor response.

The resistor-type sensor based on semiconducting metal oxide is another commonly used group of high temperature sensors which possess many advantages, such as simple configuration, easy fabrication and cost effectiveness. In resistor type oxygen sensor, a voltage is applied across the electrodes (Liu et al., 2012a) and the response of the sensor toward oxygen can be measured through changes in electrical conductance arising from alteration of the defect chemistry by chemisorption of oxygen and/or reactions between oxygen gas and sensing material (Ramamoorthy et al., 2003; Bartic et al., 2007). The bulk defect phenomena are related to oxygen ion vacancies, metal ion interstitials and vacancies, electrons and holes. The change in conductance of the material upon exposure to oxygen can be explained by the change in concentration of oxygen species in the bulk, and is a reflection of the defect structure. The predominant defects in oxide semiconductors are oxygen vacancies and their associated free change carriers. N- and P-type semiconductors have inverse directions of conductivity's change upon the interaction with oxygen, which is a very important fact for their application (Korotcenkov, 2007). For n-type semiconductor its conductivity drops upon exposure to oxygen, whereas for p-type oxide it rises.

As an important p-type semiconductor material, NiO has been receiving great research interest due to its wide band gap property, superior thermal and chemical stability. Consequently, NiO becomes a promising material in many applications such as giant magnetoresistance sensor, gas sensors, electrochemical display devices and conducting transparent electrodes (Hotovy et al., 2004; Lee et al., 2009; Gupta et al., 2012).

In this research, a bimodular oxygen sensor, including potentiometric module and resistance module, was developed through a planar sensor platform using NiO nanoparticles (NPs) thin film, which was r.f. magnetron sputter-coated on YSZ surface followed by sintering at 1100°C. The surface morphology of NiO NPs thin film was characterized by scanning electron microscope (SEM), atomic force microscope (AFM). X-ray diffraction (XRD) was applied to characterize its crystal structure and thermal stability of NiO NPs thin film before and after high temperature gas sensing. Furthermore, the oxygen sensing properties of the bimodular oxygen sensor operated at 500, 600, and 800°C was investigated under potentiometric module as well as resistance module, both of which showed sensitive, reproducible and reversible response to oxygen. The sensing mechanisms under both sensing modules were also discussed. These features demonstrate that the combination of two sensing modules into one sensor could greatly enrich the information output and has great potential in the development of novel high temperature gas sensors.

## Materials and methods

### Fabrication of bimodular sensor device

**Figure 1:**
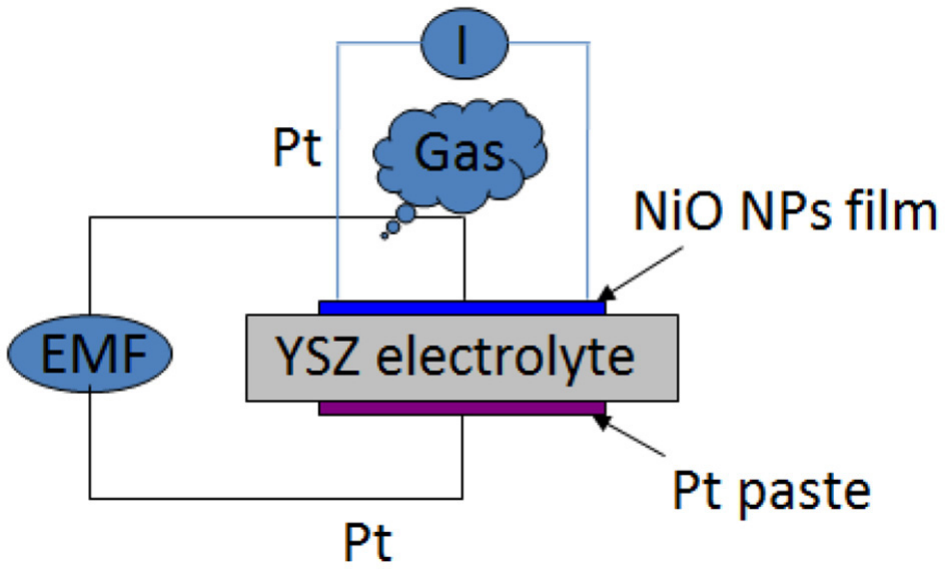
**The configuration of the developed bimodular planar high temperature gas sensor**.

An yttria-stabilized (8.0%) zirconia plate (Coating & Crystal Technology Inc., USA) with a dimension of 8 mm (length) × 8 mm (width) × 0.5 mm (thickness) was used for fabrication of the bimodular planar high temperature gas sensor device. Before loading into sputter coating chamber, the YSZ plate was chemically cleaned in order to remove dust and organic contamination (if any). Nickel oxide NPs thin films with a thickness of about 100 nm were sputtered on one side of YSZ plate by r.f. magnetron sputtering technique. Platinum (Pt) paste was then printed on the other side of YSZ plate, serving as the reference electrode in the potentiometric mode sensor. The sensor device was sintered at 1100°C for 3 h in air to increase its high temperature stability. The configuration of bimodular sensor is shown in Figure 1.

### Characterization of NiO nanoparticles thin film

The three-dimensional morphology of sputter-coated NiO NPs thin film was examined by AFM (Asylum Research MFP-3d). A JEOL 6335F field-emission SEM at 10 kV was employed to observe the morphology of sensing electrode. A Bruker XRD instrument D2 Phase with a Cu K_α_ X-ray source (λ_α_ = 1.54Å) was used to analyze the crystalline phase of NiO NPs thin film before and after high temperature oxygen sensing. The data were collected in the *2θ* range of 10–90° with a scanning rate of 12°/min.

### High temperature gas testing system

The NiO-YSZ substrate was connected with four Pt wires to realize bimodular monitoring—two Pt wires on NiO NPs thin film side used for resistance detection module and the other two Pt wires (one on NiO side and the other on Pt paste side) used for potentiometric module (Figure 1). The four Pt wires were pressed on NiO-YSZ plate by two alumina plates through mechanical force and thus built up good contact and connection with NiO-YSZ plate at its both sides. The device was then placed in a temperature-controllable furnace which connected with a mass flow controller. The two pairs of fixed Pt wires were then connected to two CHI 660D Electrochemical workstation (CH Instrument, USA) through Ni/Cr alloy wires for continuous recording of *in-situ* EMF change and resistance change of the sensing device under different oxygen concentrations and operating temperatures.

Oxygen sensing experiments were performed with a total gas flow of 1.5 L/min at different O_2_ concentrations ranging from 100 to 800 ppm and at various operating temperatures (500, 600, and 800°C). Nitrogen gas was used as the carrying gas. In a typical O_2_ sensing experiment, the sensor was first exposed to high purity nitrogen for 2 h until the baseline was stable, and then exposed to O_2_/N_2_ for 6 min, followed by high purity N_2_ to recover the sensor for 18 min (one cycle). For each oxygen concentration, two exposure/recovery cycles were conducted. The sensor circuit was subjected to 1 V DC voltage during the resistance sensing module, while for potentiometric sensing module, the EMF measurements were carried out between both sides of YSZ planar sensor with NiO NPs thin film side connected to the working electrode and Pt paste side connected to the reference electrode.

## Results and discussion

### Characterization of NiO film

**Figure 2:**
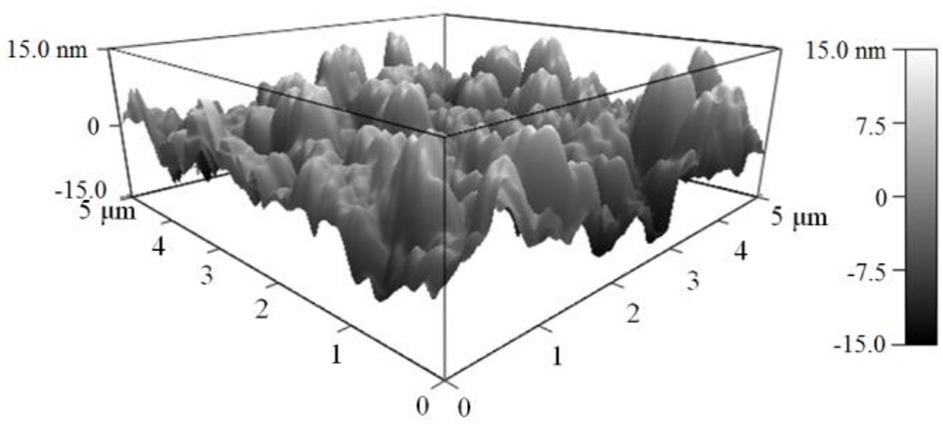
**AFM image of the surface (5 × 5 μm) of NiO nanoparticles thin film on YSZ plate after sintering at 1100°C**.

**Figure 3:**
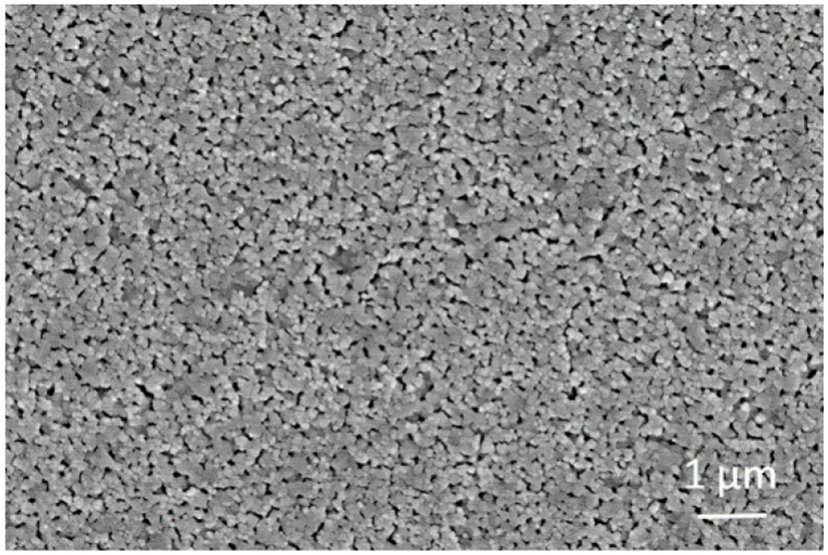
**A typical SEM image of the NiO nanoparticle thin film on YSZ plate after sintering**.

As shown in Figure 2, the AFM topography of the as-deposited NiO NPs thin film revealed that the sputter-coated NiO film, after sintering at 1100°C, consists of numerous densely-packed NPs which are in good agreement with observation in SEM image (Figure 3). The grain size of NiO was 78 ± 13 nm according to SEM characterization. Such highly porous thin film could provide large surface for gas interaction and minimize gas diffusion resistance, thus favoring subsequent high temperature gas sensing.

**Figure 4:**
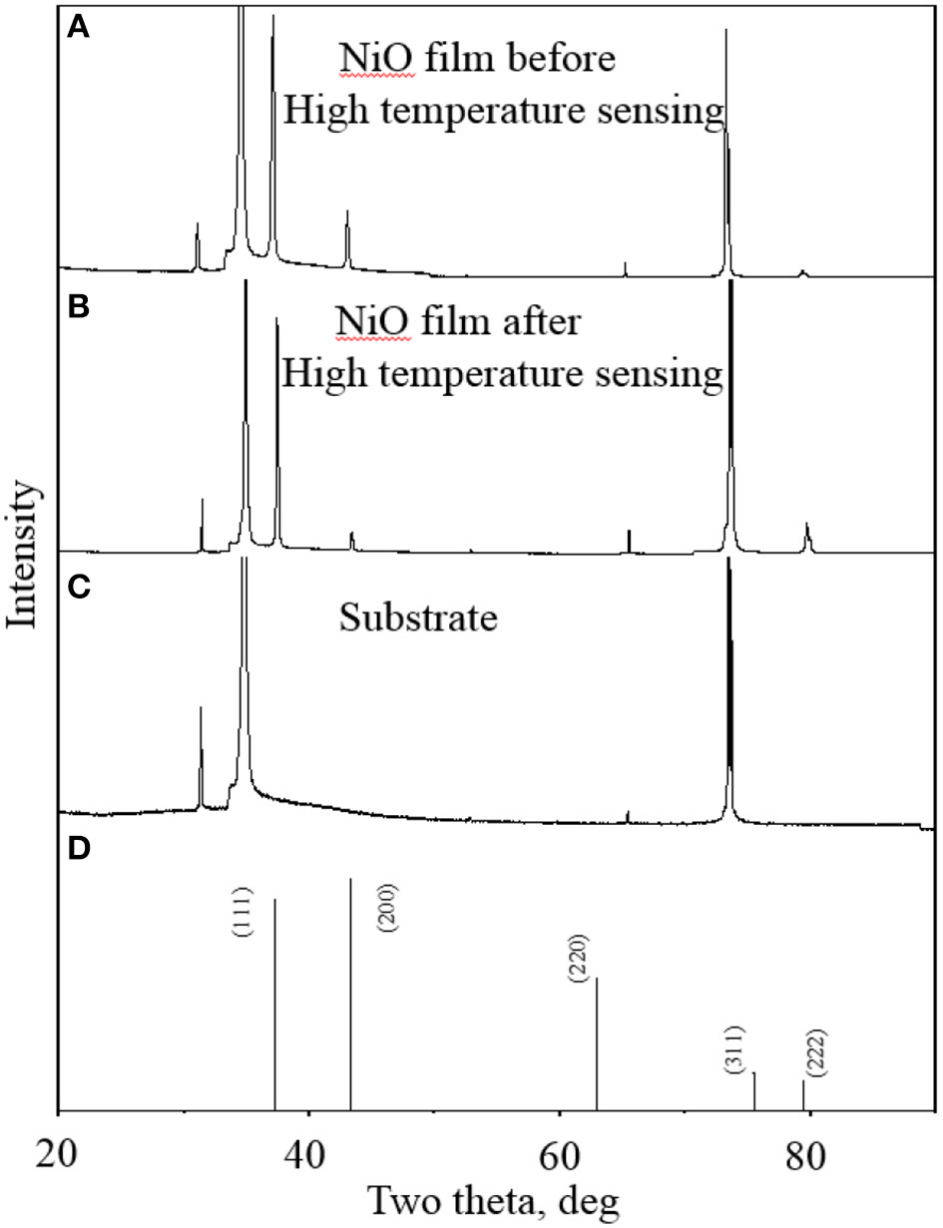
**XRD patterns of NiO nanoparticle thin films (sintered at 1100°C) before (A) and after (B) high temperature oxygen sensing experiments; (C) XRD pattern of YSZ substrate; and (D) XRD pattern of standard pattern of NiO**.

The composition and crystal structure of the as-deposited NiO NP thin film were further characterized by XRD. The XRD patterns of the NiO NPs film before and after high temperature oxygen sensing are shown in Figure 4. The formation of face-centered cubic NiO is confirmed by the diffraction peaks at 2θ values of 37.3, 43.3, and 79.4°, corresponding to (111), (200), and (222) crystal planes, respectively (Figure 4A). Other peaks can be assigned to YSZ substrate (Figure 4C). The XRD pattern (Figure 4A) matches the standard spectrum of JCPDS 4-835 (Figure 4D) except from two perspectives. Firstly, the peak corresponding to (220) is not obvious, which was consistent with other studies (Chen et al., 2005; Jang et al., 2008). Jang et al. found that there is no (220) peak for NiO after sputter-coating even with heat treatment (Jang et al., 2008). Chen et al. reported that texture coefficient (220) decreased with increasing of temperature (Chen et al., 2005). Secondly, the intensity of (111) diffraction peak is much larger than that of (200), which is different from that of JCPDS-4-835. It is known that metal oxide samples prepared in the oxide-sputtering mode showed a strong (111) diffraction peak and its intensity increased with increasing annealing temperature (Hotovy et al., 2004). Therefore, such intensity difference can be attributed to the sintering process under a high temperature of 1100°C. In order to analyze its thermal stability in high temperature sensing processes, the XRD pattern of NiO NPs thin film after high temperature oxygen sensing experiments was also investigated and presented in Figure 4B. One can see that the XRD pattern shows no peak shift compared to that before high temperature gas sensing, suggesting the superior chemical and thermal stability of NiO NPs thin film in high temperature sensing environment. Such good chemical and thermal stability endows the as-prepared NiO NPs thin film capability for high temperature O_2_ sensing.

### High temperature oxygen sensing under potentiometric module

**Figure 5:**
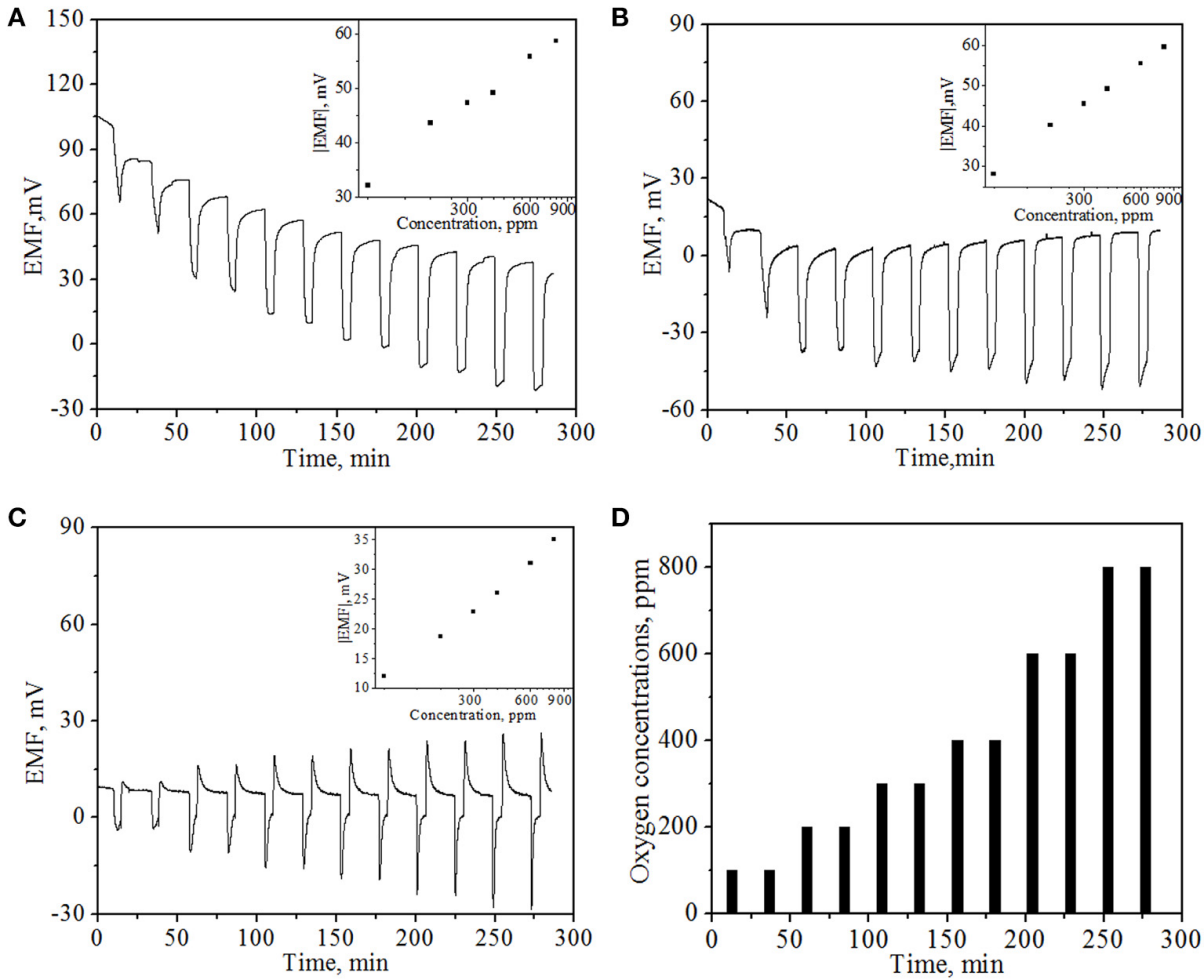
***In-situ* EMF values of potentiometric mode sensor at different operating temperatures: (A) 500°C; (B) 600°C; (C) 800°C with various oxygen concentrations**. Insets show the corresponding average EMF values of the sensor at different O_2_ concentrations. **(D)** The pattern of supplied oxygen with various concentrations in the oxygen gas sensing experiment.

The oxygen sensing property of the NiO NPs thin film was examined at operating temperatures of 500, 600, and 800°C, respectively, under potentiometric module. Figure 5 presents the typical EMF values of the sensor device as a function of time upon periodic exposure to different concentrations of O_2_ at different operating temperatures. One can see that at the operating temperatures of 500 and 600°C, the EMF values decreases rapidly upon the exposure to oxygen and displays a concentration-dependent behavior. The sensor response can be rapidly recovered using high purity N_2_ at both operating temperatures. The sensitivity to oxygen could majorly arise from the combined effects of the difference in the kinetics of oxygen reduction at TPB, catalytic activity of the electrode as a function of the concentration in the electrode, and the microstructure of the sensing electrode relative to the Pt electrode (Li and Kale, 2007). These results were in good agreement with literature report of mixed-type potentiometric oxygen gas sensor (Li and Kale, 2007). However, baseline drifting was observed at an operating temperature of 500°C, similar phenomena has also been observed by other groups (Morata et al., 2008), which suggests slower kinetics for the recovery of sensor response at lower operating temperatures. However, at 800°C, the response pattern is slightly different from those at 500°C and 600°C. Especially, the EMF value of the sensor device first recovers to a value higher than the initial EMF value and then gradually declines to the initial EMF level, indicating that a more complex mechanism may involve at higher working temperature. The dissimilar oxygen reaction on two electrodes and diffusion may play a larger role at 800°C with respect to the NiO, YSZ, and Pt surfaces in this case.

The NiO NPs thin film based potentiometric sensor can be described in the form of electrochemical cell as shown below:

In the base gas, N_2_, NiO|YSZ| Pt, N_2_
In the sample gas, O_2_+N_2_, NiO|YSZ| Pt, O_2_+N_2_

The oxygen electrochemical reaction (O_2_ + 4*e*^−^ ⇔ 2O^2−^) is equilibrating. However, the reactions take place at different rates on the dissimilar electrodes, and thus a potential difference is generated, which leads to a measurable EMF variation. Besides electrochemical reaction mechanism, Bartolomeo et al. proposed adsorption mechanism (Di Bartolomeo et al., 2004), in which the variation of EMF is consistent with the changes in resistance induced by chemisorption of oxidizing or reducing gas on n-type or p-type semiconductors related to electrocatalytic activity. NiO is a p-type semiconductor at high temperature. When NiO is exposed to oxygen at the operating temperature, the resistance of NiO decreases and thus leads to the decrease of EMF value.

The insets of Figure 5 present the plots of average EMF value vs. O_2_ concentrations at corresponding operating temperatures. It can be observed that EMF value of the sensor device exhibits a linear dependence on the logarithm of oxygen concentrations, as expected from well-established Nernst equation (Ramamoorthy et al., 2003; Moos et al., 2009). In addition, it can be observed from Figure 5 that in general, the sensor response to the same oxygen concentration follows the decline trend with the increase of temperature. For example, the EMF values were 47.3, 45.5, and 22.8 mV for 300 ppm O_2_ concentration at 500, 600, and 800°C, respectively. This phenomenon was compatible with previous reports (Di Bartolomeo et al., 2004; Fergus, 2007a; Plashnitsa et al., 2008) and also can be well-explained by aforementioned adsorption mechanism (Di Bartolomeo et al., 2004). In general, similar mixed potential difference response tends to decrease with increasing temperature because of following reasons. Chemical reactions tend toward equilibrium with increasing temperatures, thus a sensor response heavily relies on kinetically limited response. The increase of temperature would lead to a faster kinetics for gas redox reaction on both electrodes, resulting in a reduction of potential difference between them. Therefore, mixed potential type sensor has one drawback that cannot be applied in very high temperature environment, which greatly limits its broader applications as high temperature gas sensors.

### High temperature oxygen sensing under resistance module

**Figure 6:**
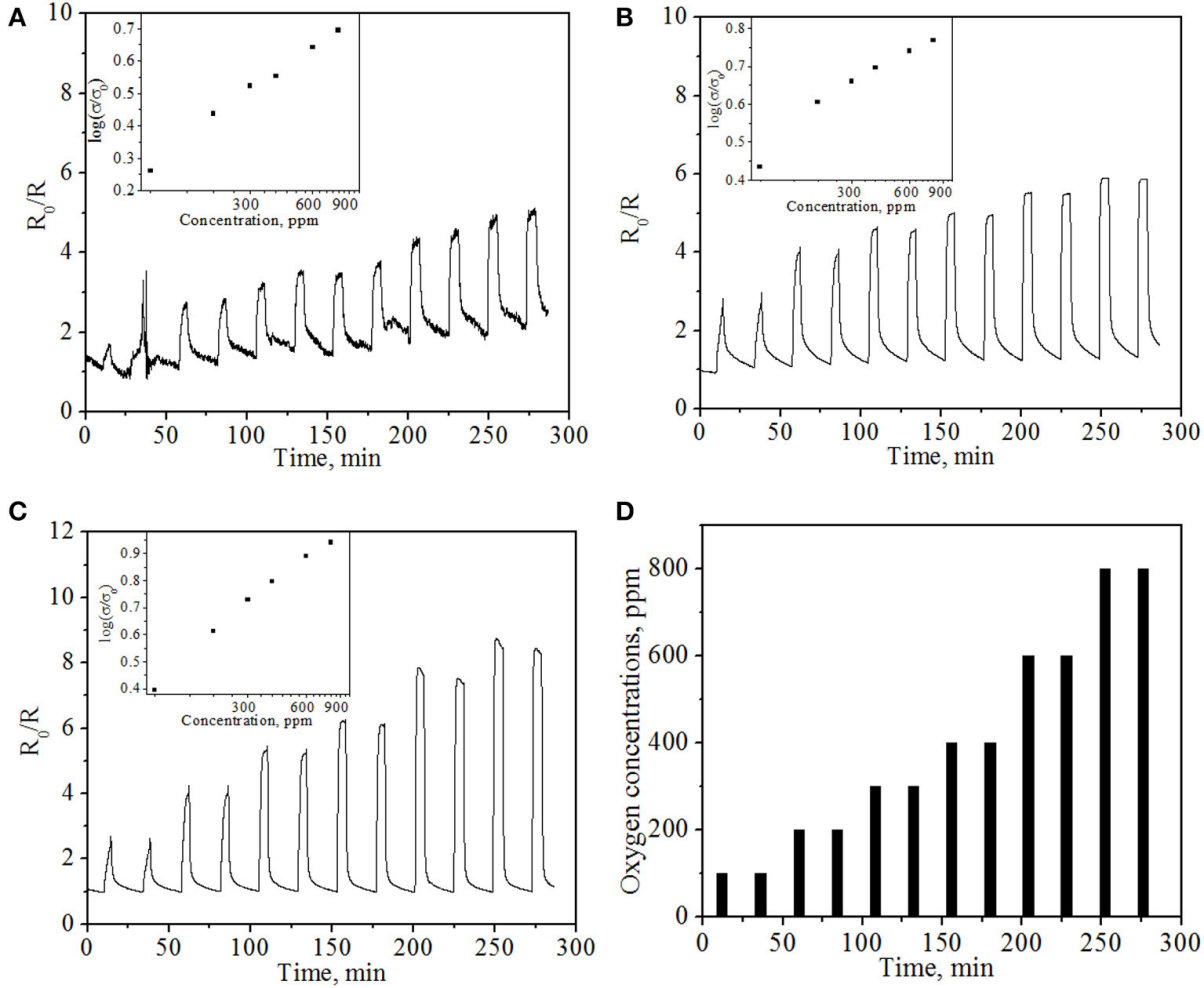
***In-situ* sensing response of resistance type sensor at different operating temperatures: (A) 500°C; (B) 600°C; (C) 800°C with various oxygen concentrations**. Insets show the average of normalized electrical conductivity of the sensor vs. oxygen concentration. **(D)** The pattern of supplied oxygen with various concentrations in the oxygen gas sensing experiment.

The O_2_ sensing properties of the sensor device were further examined at operating temperatures of 500, 600, and 800°C, respectively, under resistance module (the resistance of NiO film). The applied bias in the sensing experiments was 1 V. The current in the sensor was continuously measured and the electric resistance of the sensor was calculated by applying Ohm's Law (R = V/I). R_0_/R, where R is the real-time sensor resistance with change of gas concentrations and R_0_ is the initial sensor resistance in N_2_, were employed as the sensor response. Figure 6 represents typical electrical responses of the sensor device as a function of time upon periodic exposure to gaseous oxygen balanced in high purity N_2_. Upon exposure to oxygen, the NiO NPs thin film shows reproducible, sensitive and concentration-dependent resistance change to oxygen, suggesting that NiO NPs thin film is a good sensing material for resistance-based oxygen detection at high temperature. By purging with nitrogen, the response of sensor can be recovered, indicating the quick desorption of oxygen molecules from the NiO NPs and the good reproducibility of NiO NPs thin film based oxygen sensor. The good performance and response/recovery can be attributed to the highly porous structure of sensing material and nanoscale size of NiO, in which porous structure allows the free access of oxygen molecules to NiO with reduced diffusion resistance while nanoscale size allows fast chemisorption and desorption of gas molecules (Liu et al., 2012a). In addition, no obvious baseline drifting was observed for repeated operation, indicating the good stability.

At fixed operating temperatures, the sensing response (R_0_/R) increases with oxygen concentration from 100 to 800 ppm. For example, at an operating temperature of 600°C, the response increases from 4.03 to 5.89 when O_2_ concentrations increase from 200 to 800 ppm. At fixed oxygen concentration (e.g., 300 ppm oxygen), the sensing response increases from 3.33 to 4.57 to 5.34 when the temperature increases from 500°C to 600°C to 800°C, respectively, which is different from the observation for previous potentiometric module. This result implies that resistance sensing module may have greater potential to be applied in higher temperature environment compared to potentiometric module. Combining two detection modes into one sensor, one can realize a wide operating temperature window and also greatly enrich the information output.

At high temperatures, NiO exhibits a p-type semiconductor behavior. First, NiO can be readily made slightly oxygen-rich and thus its crystal must have a population of point defects (Tilley, 2011). This can be considered as a reaction of oxygen gas with stoichiometric NiO, assuming that extra oxygen extends the crystal by adding extra oxygen sites. As reported earlier (Tilley, 2011), the following defect reaction can occur:

(1) $$\frac{1}{2}{\text{O}}_{2}(\text{NiO})↔{\text{O}}_{0}+{\text{V}}_{\text{Ni}}^{2^{\prime }}+2{\text{h}}^{•}.$$

The creation of each cation vacancy is accompanied by the creation of a hole. For NiO, ionic crystal structure is assumed, and then a p-type behavior would be expected. Consequently, with the increase of oxygen concentrations, the above equation shifts to the right and generates more holes, thus leading to an increase of electrical conductivity, in good agreement with the experimental results. On the other hand, removal of O_2_ could shift the equation to the left side, and then bring the conductivity back to the original level and regenerate the sensor.

A relationship between the oxygen partial pressure and the electrical conductivity of an oxide (Ramamoorthy et al., 2003; Liu et al., 2012b) can be further represented by

(2) $$\sigma =A\exp\left(-E_{a}/kT\right)P_{O_{2}}^{m}$$

where σ is the electrical conductivity, *A* is a constant, *E*_*a*_ is the activation energy for conduction, *P*_*O*_2__ is the oxygen partial pressure, and *m* is a parameter determined by both the type of the carrier (n or p) and the defects (e.g., oxygen vacancy) in the semiconductor. For p-type oxide, *m* is a positive value. The normalized electrical conductivity of NiO NPs thin film vs. oxygen concentration at different temperatures is shown in the insets of Figure 6. At a fixed operating temperature, the normalized conductivity is proportional to the logarithm of oxygen concentration, which was consistent with the aforementioned equation as well as the experimental results. However, compared to the theoretical value *m* = 1/6 to 1/4 in σ ∝ P(O_2_)^m^ (Stubican and Carinci, 1998; Birks et al., 2006), the obtained *m* values for NiO NPs thin film are 0.42, 0.27, and 0.54 at 500, 600, and 800°C, respectively. The value of *m* at 600°C is very close to the theoretical value ¼, while the values at 500 and 800°C are slightly larger than the predicted value. Similar phenomenon has been observed in literature, in which *m* value was reported to be close to 0.5 due to the potential contribution of hole concentration on the surface (Palombari, 2003). The observed high *m* values in this study may be ascribed to the influence of donor-acceptor coupling, high hole concentration on the surface, significant contribution of ionic conductivity, and/or grain boundary diffusion associated with nanoscale NiO (Liu et al., 2012b). It is also possible that YSZ electrolyte plays a role in *m* value, since YSZ is a good ionic conductor at elevated temperatures. Typically, the higher the *m* value, the higher the oxygen response of the sensor. Therefore, the high oxygen response of NiO NPs film obtained in this study indicates that NiO NPs film is a good candidate in the construction of bimodular gas sensor for the detection of oxygen in high temperature environment. According to Equation 2, the calculated activation energy is 12.4 ± 1.3 kJ/mole, which is also in good agreement with reported values of 10.4 ~ 13.8 kJ/mole and 12.5 kJ/mole (Hakim et al., 2009; Guziewicz et al., 2011).

## Conclusions

In summary, NiO NPs thin film with good thermal stability was successfully coated on YSZ substrate by r.f. magnetron sputter-coating followed by sintering at 1100°C. A novel bimodular planar O_2_ sensor based on the as-prepared NiO/YSZ was then fabricated and applied for high temperature oxygen detection under two modules—potentiometric and resistance. Both potentiometric module and resistance module of the developed bimodular sensor exhibit sensitive, reproducible and reversible response to oxygen. The sensing mechanisms for both modules were also discussed. Integration of two sensing modules into one sensor could greatly enrich the information output and would open a new venue in high temperature gas sensor development.

## Author contributions

Xiangcheng Sun performed the experiments and analyzed the data. Xiangcheng Sun and Yu Lei conceived and designed the experiments, performed the data analyses and wrote the manuscript. Yixin Liu contributed to the resistance module experimental design and explanation. Haiyong Gao participated in NiO film preparation and AFM characterization. Pu-Xian Gao and Yu Lei coordinated and financed the project. All the authors commented on the manuscript.

### Conflict of interest statement

The authors declare that the research was conducted in the absence of any commercial or financial relationships that could be construed as a potential conflict of interest.
